# Nkrp1 Family, from Lectins to Protein Interacting Molecules

**DOI:** 10.3390/molecules20023463

**Published:** 2015-02-17

**Authors:** Daniel Rozbeský, Ljubina Ivanova, Lucie Hernychová, Valéria Grobárová, Petr Novák, Jan Černý

**Affiliations:** 1 Institute of Microbiology, v.v.i., Academy of Sciences of the Czech Republic, Vídeňská 1083, Prague 414220, Czech Republic; E-Mails: rozbesky@gmail.com (D.R.); ivanova@biomed.cas.cz (L.I.); lucie.hernychova@biomed.cas.cz (L.H.); 2 Department of Biochemistry, Faculty of Science, Charles University, Hlavova 8, Prague 212843, Czech Republic; 3 Department of Cell Biology, Faculty of Science, Charles University, Viničná 7, Prague 212843, Czech Republic; E-Mail: valeria.grobarova@natur.cuni.cz

**Keywords:** C-type lectin-like receptors, lectin, Clr, CTLD, NK cells, Nkrp1, saccharide

## Abstract

The C-type lectin-like receptors include the Nkrp1 protein family that regulates the activity of natural killer (NK) cells. Rat Nkrp1a was reported to bind monosaccharide moieties in a Ca^2+^-dependent manner in preference order of GalNac > GlcNAc >> Fuc >> Gal > Man. These findings established for rat Nkrp1a have been extrapolated to all additional Nkrp1 receptors and have been supported by numerous studies over the past two decades. However, since 1996 there has been controversy and another article showed lack of interactions with saccharides in 1999. Nevertheless, several high affinity saccharide ligands were synthesized in order to utilize their potential in antitumor therapy. Subsequently, protein ligands were introduced as specific binders for Nkrp1 proteins and three dimensional models of receptor/protein ligand interaction were derived from crystallographic data. Finally, for at least some members of the NK cell C-type lectin-like proteins, the “sweet story” was impaired by two reports in recent years. It has been shown that the rat Nkrp1a and CD69 do not bind saccharide ligands such as GlcNAc, GalNAc, chitotetraose and saccharide derivatives (GlcNAc-PAMAM) do not directly and specifically influence cytotoxic activity of NK cells as it was previously described.

## 1. Introduction

Natural killer (NK) cells are an essential component of the innate immunity system that play a key role against virally infected or tumor cells. The specificity of NK cells for target cells is determined by a sophisticated repertoire of activating and inhibitory receptors expressed on the surface of NK cells [1]. Besides the eradication of a variety of different pathological cells, NK cells produce several classes of immunoregulatory cytokines, which help to shape the immune response. Interactions between these molecules are involved in maintaining homeostasis within the immune system [2]. Furthermore, NK cells are also involved in transplantation immunity [3], autoimmunity [4], allergic diseases [5] or reproduction [6].

The cytolytic activity of NK cells is determined by a dynamic interplay between activating and inhibitory signals [7,8,9]. According to the missing self-recognition, the inhibitory receptors recognize ligands, such as MHC I molecules, which are broadly expressed by healthy cells but their expression is significantly downregulated during pathological circumstances. On the other hand, according to the induced self-recognition, activating receptors recognize ligands, such as MICA [10], which are minimally expressed by healthy cells but their expression is strongly upregulated during cellular stress, and infectious or tumorigenic processes.

NK receptors belong to two distinct structural classes: the immunoglobulin-like receptors and the C-type lectin-like receptors. The first group includes Killer Cell Immunoglobulin-Like Receptors (KIR), Leukocyte Immunoglobulin-Like Receptors (LILR) and Natural Cytotoxicity Receptors (NCR) protein families, the second one Ly49, NKG2D, CD94/NKG2 and Nkrp1 protein families.

The Nkrp1 protein family contains an important set of both activating and inhibitory receptors. The first identified Nkrp1 receptors were originally classified as members of the C-type animal lectin family [11,12]. Moreover, the ability to bind saccharides was extended to other Nkrp1 receptors and several high affinity saccharide ligands with antitumor potential were synthesized. However, due to irreproducibility of binding experiments the saccharide binding activity of Nkrp1 receptors became a matter of controversy and most of the NK receptor research field dismissed the saccharide binding properties of Nkrp1. This review is focused on Nkrp1 protein family and their biological functions. Particularly, we discuss discrepancies concerning Nkrp1 ligand binding specificity accumulated during 20 years of the research in the field.

## 2. Nkrp1 Family

The first cell-surface antigen specific for mouse NK cells was described in 1977 [13]. Later, it was named NK1.1 and classified as a member of the Nkrp1 family [14], Nkrp1c [12,15]. Since then several other *Nkrp1* (*Klrb1*) genes have been identified.

In the mouse genome, members of the *Klrb1* family (*Klrb1a*, *b/d*, *c*, *e*, *f* and *g* [12,16,17,18] encoding Nkrp1a, b/d, c, f and g proteins) are located in the Natural Killer Cell (NKC) gene complex and map to the chromosome 6 [19]. *Klrb1b* and *d* genes represent two divergent alleles of the same locus and *Klrb1e* is a pseudogene [16]. Four *Klrb1* genes located on chromosome 4 [20] were defined in rat: *Klrb1a* [21,22], *b/c* [20,23], *f* [24] and *g* [17] (*Klrb1b* and *c* are also divergent alleles [17]) encoding Nkrp1a, b/c, f and g proteins. There is only one *KLRB1* gene encoding NKRP1A in the human genome, which is located on chromosome 12 [15]. Besides NK cells, Nkrp1 receptors are also expressed on the surface of NKT cells, T lymphocytes and dendritic cells [24,25].

## 3. Structure of Nkrp1 Receptors

From a structural point of view, Nkrp1 receptors are type II transmembrane proteins containing an N-terminal cytoplasmic domain, a transmembrane region, and an ectodomain with the C-type lectin-like domain (CTLD), which is connected by a stalk region to the transmembrane segment. Several members of the Nkrp1 family have been demonstrated to exist as disulfide linked homodimers [11,12,26], in which cysteines in the stalk region mediate intermolecular disulfide bonds. Nevertheless, the existence of Nkrp1 proteins as disulfide linked homodimers requires further investigation as some members of the Nkrp1 family show variability in the number of cysteines in the stalk region [27].

**Figure 1:**
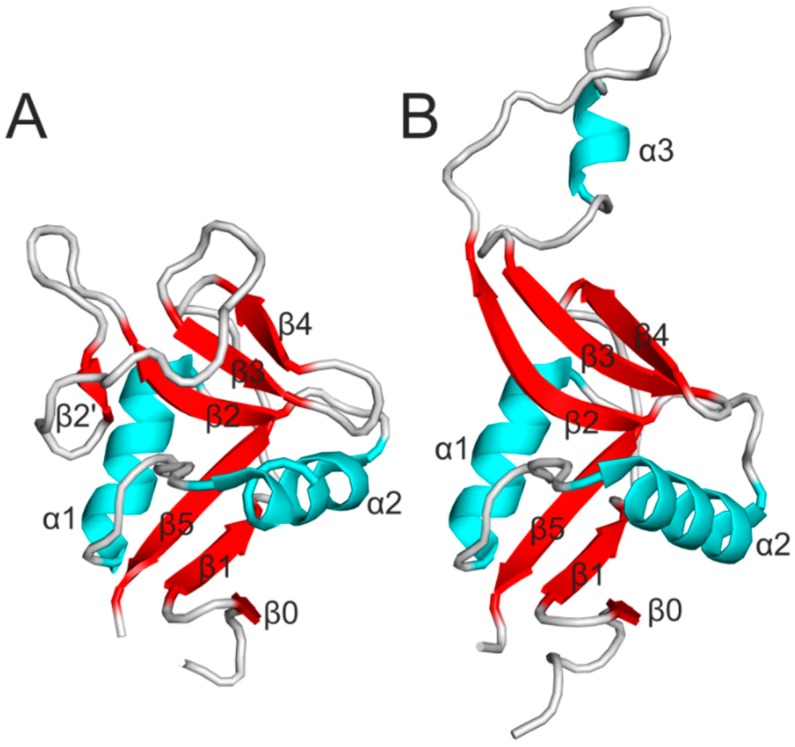
(**A**) Ribbon diagram of a typical CTLD. Secondary structure elements are labeled. α-helices are cyan, β-strands red and loops are gray; (**B**) Structure of the mouse Nkrp1a with a loop region pointing away from the central core.

In addition, the CTLD shares structural homology with the C-type lectin domain, but contrary to the C-type lectin domain, CTLD lacks the residues involved in calcium binding and recognizes other ligands than saccharides. The CTLD consists of two α-helices (α1 and α2) and two antiparallel β-sheets (Figure 1A). Numbering of secondary structural elements is usually based on rat MBP-A [28]. In most of the known three-dimensional structures of CTLD, the two β-sheets are formed by β0, β1 and β5 strands, and β2, β2’, β3 and β4 strands, respectively. Four cysteine residues, which are the most conserved residues in CTLD, adopt a disulfide pattern of C1-C4 and C2-C3. On the other hand, arrangement of the loops connecting the secondary structural elements differs to a large extent between the particular CTLDs [29].

In terms of Nkrp1 receptors, initial work on their structure demonstrated that Nkrp1 receptors adopt the fold typical for the CTLD with increased flexibility of a long loop region, which corresponds to the region between strands β2 and β3. Moreover, the sequence differences in the loop suggested that the long loop region might encode the ligand specificity [30]. Indeed, the crystal structure of mouse Nkrp1a, which is the only known structure among Nkrp1 receptors so far, revealed that Nkrp1a adopts a fold similar to the CTLD with the exception of the extended long loop region pointing away from the CTLD (Figure 1B). In the crystal, the extended loop tightly interacted with a neighboring loop of another molecule using a domain swapping effect [31]. The position of the extended loop pointing away from the CTLD has not been described in any NK receptors. However, this conformation has been observed in other C-type lectin-like proteins important in the immune system such as the macrophage mannose receptor [32] and in snake venoms [33]. On the other hand, structural analysis of mouse Nkrp1a and Nkrp1c in solution using mass spectrometric techniques unveiled that the loop region was not extended in solution, but instead collapsed and closely interacted with the core of the molecule [34,35]. Moreover, the recently determined structure of NKp65 in complex with its ligand KACL, which is the first report of CTLD recognition by another CTLD, suggests that the extended loop of Nkrp1a is sterically incompatible with the NKp65 mode of ligand binding and probably represents a crystallization artifact [36].

## 4. Signaling

Signals from many different NK receptors enter the cell simultaneously and need to be integrated. Through the inhibitory receptors, signaling is commonly mediated via tyrosine phosphorylation of an Immunoreceptor Tyrosine-based Inhibitory Motif (ITIM), which is present in the cytoplasmic part of the NK receptor and is a component of mouse and rat Nkrp1b/d [37,38] or Nkrp1g receptors [18].

Phosphorylation of Immunoreceptor Tyrosine-based Activation Motifs (ITAM), typically in adaptor proteins (e.g., FcRγ or CD3ζ) is crucial for signal transduction of activating receptors. Receptors usually contain positively charged amino acids in their transmembrane region interacting with negatively charged amino acids in associated adaptors [39,40]. The most important motif for a subsequent signaling cascade in the cytoplasmic domain of all murine NK receptors, except the Nkrp1g isoform, is CxCP (Cys-X-Cys-Pro), which represents a binding site for a kinase of the Src family, Lck [41]. Lck phosphorylates tyrosines in ITAM/ITIM sequences as was described for mouse Nkrp1c and Nkrp1b [40]. In case of an activating receptor, phosphorylation leads to recruitment of the Syk kinase [42], that triggers a downstream cascade. Conversely, phosphorylation of ITIM creates a docking template for the phosphatase SHP-1 [43,44], which dephosphorylates substrates of kinases functioning in activating pathways [45].

Human NKRP1A represents an exception in such signaling. Although the receptor has an inhibitory function, it does not possess an ITIM sequence or Lck binding motif in its cytoplasmic domain. Signaling via human NKRP1A involves association with acid sphingomyelinase, production of ceramide, and PI3K-dependent activation of the Akt and ERK pathways [46].

## 5. Saccharide Ligands of Nkrp1—20 Years of Controversy

The ligands for rat Nkrp1 have been a matter of controversy for 20 years. The first report showing Nkrp1 as a lectin binding monosaccharides was published by Bezouska *et al*., in 1994. The binding of monosaccharides was reported to be strongly Ca^2+^-dependent in preference order of GalNac > GlcNAc >> Fuc >> Gal > Man [47]. These results were extended by the identification of high affinity oligosaccharides of the blood group family, the ganglio family and glycosaminoglycans with IC_50_ in the range 10^−9^–10^−12^ M. These high affinity oligosaccharide ligands localized at the cell membrane were reported to lead to cytolysis of tumor cells suggesting a new and particularly promising antitumor therapy [48].

However, a correction was published in 1996 by six out of the ten original authors, who wished to retract that article owing to their inability to reproduce the results [49]. Furthermore, Nkrp1 monosaccharide binding activity was re-evaluated in 1999 as well, showing no data indicating specific binding to any of the monosaccharides as described in the previous report [50].

Despite all the controversies and contradictions, many studies have been accumulated using the binding paradigm from 1994 in data interpretations. Simultaneously, other ligands for Nkrp1 were described. New glycomimetics (saccharide molecules with some variations in the structure, modifying biological properties) on GlcNAc basis were synthesized since GlcNAc had been shown in previous articles to be the best ligand among the monosaccharides tested [47]. Very high affinity was observed for a number of chitooligomers, particularly chitotetraose, and thus several chitotetraose derivatives were synthesized and reported as high affinity ligands of Nkrp1 [51,52,53,54,55]. A next generation of glycomimetics with strong antitumor response was based on highly branched GlcNAc-terminated glycoclusters such as GlcNAc coated octavalent glycodendrimers (PAMAM-GlcNAc_8_). In addition, the considerable immune response was explained by a multivalency effect of these derivatives [56,57,58,59].

Further studies revealed disaccharides such as ManNAc(1→4)Glc [60] or monovalent and bivalent LacdiNAc as ligands with exceptional affinity to Nkrp1 [61,62]. Moreover, several negatively charged oligosaccharides and glycosides [63,64,65], or deoxynorijimycin with its hexosaminyl derivatives [66], as well as glycosyl-1H-1,2,3-triazoles [67] were demonstrated as effective Nkrp1 ligands too. Furthermore, series of calixarenes substituted with GlcNAc were reported as high affinity ligands of Nkrp1 and described as a next generation of saccharide derivatives with a strong antitumor effect [68].

Direct binding between Nkrp1a and saccharide ligands was extended by biological assays in mouse models. Indeed, treatment with PAMAM-GlcNAc_8_ or GlcNAc-coated calixarene dendrimer was reported to reduce tumor growth in melanoma-bearing C57BL/6 mice and prolonged survival time of experimental animals in several studies. Additionally, high levels of IgG as a result of increased antibody-dependent cell-mediated cytotoxicity (ADCC) and upregulated synthesis of IFN-γ by NK cells was observed [69,70,71].

Besides the rat and mouse Nkrp1 receptors, interactions of the synthesized saccharide ligands were tested also with human CD69, a C-type lectin-like receptor structurally similar to Nkrp1, and most of them showed binding patterns similar to rat Nkrp1 [72,73,74,75]. The majority of the above mentioned descriptions of the Nkrp1 ligand specificity were provided by a limited number of laboratories, with some methodologies performed and interpreted only by a single scientist.

In 2012 Karel Bezouska was investigated by the Ethical committee of the Institute of Microbiology and the Charles University in Prague. After investigation the committee announced that Bezouska had repeatedly and for several years committed scientific misconduct, and asked the coauthors of all respective papers to re-evaluate their experiments and interpretations [76].

In the context of later findings, the binding activity of Nkrp1 described before was re-examined over the past two years. The interaction between rat Nkrp1a and multiple ligands was re-evaluated using two independent biophysical techniques, isothermal titration calorimetry and NMR titration. Though the same experimental conditions (buffer, temperature, calcium concentration, a positive and a negative control sample *etc.*) were used as described before, no binding was detected [77]. Moreover, the interaction analysis using a neoglycoconjugate binding assay revealed no affinity between rat Nkrp1a or mouse Nkrp1c and the various neoglycoproteins such as *N*-acetylglucosamine_36_BSA. Furthermore, no significant changes in serum cytokine patterns relevant to anti-cancer treatment, or changes in *Klrb1* gene expression were observed in animal models after injection with PAMAM-GlcNAc_8_ [78]. These findings raised serious doubts about the credibility of several previous papers. Indeed, most of them have been already retracted while the others are still in the retraction process.

However, the lack of interactions with the tested saccharide samples does not imply, that Nkrp1 might not bind other saccharide moieties. Interestingly, several C-type lectin-like receptors, including the inhibitory receptors Ly49A and C, have been reported to recognize sulfated polysaccharides [79]. In addition, glycosylation plays an important role in NK cell activation and regulation of their effector functions. Hartmann *et al*. published that glycosylation of the stalk domain of the NK receptor NKp30 influences receptor-ligand interactions and intracellular signaling via an adaptor protein [80]. Similarly, binding of inhibitory receptors Ly49 to MHC class I molecules is affected by glycosylation, but contrary to NKp30, glycosylation of Ly49 receptors lowers the affinity to their ligands [81]. Further, altered glycosylation on the cell surface of cancer cells may result in modulation of surface expression of NKD2D ligands indicating that the surface expression of MICA/B is dependent on *N*-linked glycosylation [82].

## 6. The Clr Ligands

**Figure 2:**
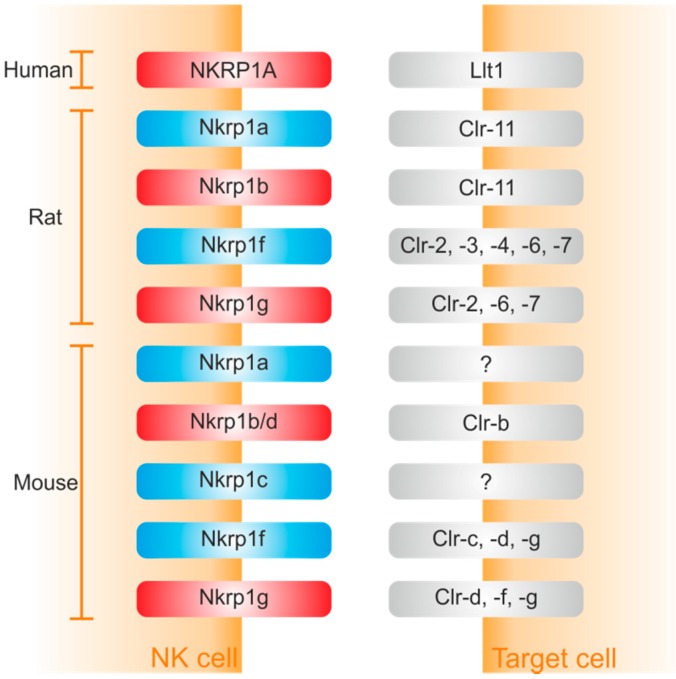
Human, rat and mouse Nkrp1-Clr interactions showing the combinatorial nature of the Nkrp1 repertoire. Inhibitory receptors are in red; activating in blue.

Meantime, protein ligands for several members of the Nkrp1 receptor family have been identified (Figure 2). They represent the C-type lectin-related (Clr) protein family, alternatively known as the osteoclast inhibitory lectin (Ocil). Intriguingly, Clr ligands share 40% amino acid identity with the CTLD of CD69 [83,84] and are encoded by genes (*Clec2*) intermingled within NKC (Natural Killer Cell gene complex) with *Klrb1* genes encoding their receptors [26].

In mouse, binding pairs include Nkrp1b/d:Clr-b, Nkrp1f:Clr-c,-d,-g, and Nkrp1g:Clr-d,-f,-g; ligands for Nkrp1a and Nkrp1c remain still elusive [18,26,31,34,35,41,83,85,86,87]. In rat, Nkrp1a and Nkrp1b recognize Clr11, whereas Nkrp1f and Nkrp1g recognize Clr-2,-3,-4,-6,-7 and Clr-2,-6,-7, respectively. Interestingly, both mouse and rat Nkrp1f and Nkrp1g show high promiscuity with an overlapping set of ligands [17].

In human, the only receptor in the Nkrp1 family, NKRP1A, binds LLT1, which is structurally similar to mouse Clr proteins [88,89]. Surface plasmon analysis of the interaction revealed that the extracellular domain of NKRP1A binds LLT1 with fast kinetics and low affinity with K_d_ = 48 µM. Binding is entropically and enthalpically driven with a small heat capacity. In addition, these binding properties are typical features for cell-cell recognition molecules. Experiments employing mutagenesis of the proteins indicated a new structural model suggesting a dimer-dimer interaction [86]. Nevertheless, the crystal structure of any Nkrp1:Clr complex remains to be determined.

## 7. Biological Relevance of Nkrp1:Clr Interactions

In general, inhibitory C-type lectin-like receptors interact with MHC class I or MHC class I-like molecules. Classical MHC I molecules are recognized by members of the mouse Ly49 receptor family, whereas non-classical MHC I molecules associate with the heterodimeric CD94/NKG2 receptor [90,91,92] and with the activating NKG2D receptor, respectively [93].

Interestingly, inhibitory receptors Nkrp1b/d recognize MHC-independent ligands. Clr-b, which is expressed on most hematopoietic and several non-hematopoietic cell types, displays an expression pattern comparable to classical MHC I antigens [94]. Moreover, it is downregulated in poxvirus-infected [95] and tumor cells, which contributes to higher susceptibility of target cells to NK cell-mediated cytotoxicity, supporting the “missing self” hypothesis [85].

Additionally, a rapid downregulation of Clr-b at both RNA and protein levels under chemotherapy-induced genotoxic stress has been shown in some leukemia cells resulting in decreased NK-cell inhibition mediated by the Nkrp1b:Clr-b receptor-ligand pair. Further on, the levels of MHC class I molecules remained normal [96], suggesting that the functional effect of the Nkrp1b:Clr-b interaction is strictly regulated by Clr-b expression levels [25,85].

In the mouse interaction between Nkrp1f and Clr-g resulted in stimulation of TCR/CD28-mediated T cell proliferation and IL-2 production in *in vitro* and *in vivo* experiments, respectively [97]. However, since only basic studies about the surface expression of both interaction partners and their specific binding are available, further detailed functional analysis is required.

Moreover, the role of Nkrp1b:Clr-b interactions in innate immunity and viral infections has been investigated. It was described that rat cytomegalovirus (RCMV) has developed a mechanism to avoid NK cell killing through the expression of a viral protein RCTL on the surface of an infected cell; RCTL is a homolog of rat Clr-b interacting with Nkrp1b on NK cells [98]. In mice with a knocked-out *Ly49H* gene, encoding another NK cell activating receptor, mouse cytomegalovirus (MCMV) infection causes extensive viral expansion and initiates effector cytokine secretion [99]. Moreover, in some rat strains, Nkrp1b has numerous allelic variations which abort the viral protein-Nkrp1b interaction and support host Nkrp1b:Clr-b recognition [23,38].

The NKRP1A:LLT1 interaction in human causes inhibition of cytotoxicity and IFN-γ secretion in NKRP1^+^ NK cells [89]. In contrast, LLT1 engaging NKRP1A on T cells provides T cell costimulation and increases cytokine production [100]. Furthermore, expression of LLT1 on TLR-stimulated plasmacytoid dendritic cells and monocyte-derived dendritic cells might explain the signal transduction pathways between NK cells and dendritic cells during an immune response to pathogens [101]. While the NKRP1A:LLT1 interactions on NK and T cells have different effects *in vitro*, the immunological relevance of this engagement *in vivo* is not well understood. A recent study has shown the connection between respiratory-virus infection and proinflammatory cytokine production, where LLT1 expressed on epithelial cells may represent a part of a regulatory feedback mechanism [102].

Generally, structural analysis is obligatory to understand the functions of proteins or protein-protein interactions. Nonetheless, structural characterization of the molecular details of the Nkrp1:Clr interaction is at the beginning. Until now there is only a limited number of relevant receptor or ligand structures solved, namely for mouse Nkrp1a [31,103] and Clr-g proteins [87]. The first crystal structure of the CTLD in complex with another CTLD, the NKp65:KACL complex, has been suggested to be a model structure for the rest of the known rodent Nkrp1:Clr and human NKRP1A:LLT1 receptor-ligand pairs, respectively [36].

## 8. Conclusions

Nkrp1 receptors were originally classified as members of the C-type animal lectin family with well documented ligand binding specificity. There were several reasons for this notion, e.g., sequence homology to well described lectin molecules or striking and well documented data published in high ranking journals. It was temping for the NK-cell receptor community to extrapolate data from one model protein (rat Nkrp1a) to a broader set of molecules encoded in different species (mouse, human), establishing a paradigm in the field. It is not surprising that lectin-based interpretation of the primary data led to misleading results, which accumulated during the years, even in the situation when early after publishing the seminal article in Nature a correction and publications questioning the relevance and the reproducibility of the results, appeared. Over the years more and more data challenging the lectin-based concept—mutations in the calcium binding site, originally saccharide binding site, turned out to better fit a protein-recognition module and finally, specific protein ligands for members of the Nkrp1 family were found and protein-protein interactions were characterized in molecular details. Whatever kind of a mistake there was at the beginning, it sent at least part of the researchers in a wrong direction for decades. It is satisfactory that the evidence coming from different sources finally results in the change of the paradigm and clarifies the field of NK-cell receptor biology.

## Abbreviation

**CTLD**: C-type lectin-like domain
**GalNAc**: 2-acetamido-2-deoxy-d-galactopyranose
**GlcNAc**: 2-acetamido-2-deoxy-d-glucopyranose
**ITAM**: immunoreceptor tyrosine-based activation motif
**ITIM**: immunoreceptor tyrosine-based inhibitory motif
**LacdiNAc**: 2-acetamido-2-deoxy-β-d-galactopyranosyl-(1→4)-2-acetamido-2-deoxy-d-glucopyranose
**ManNAc**: 2-acetamido-d-mannopyranose
**NKC**: NK cell gene complex
**PAMAM dendrimer**: polyamidoamine dendrimer
